# Exploring the Anti-Leukemic Effect of the Synthetic Retinoid ST1926 on Malignant T Cells: A Comprehensive Proteomics Approach

**DOI:** 10.3390/ijms26104651

**Published:** 2025-05-13

**Authors:** Mona Goli, Vishal Sandilya, Botheina Ghandour, Hiba El Hajj, Firas Kobeissy, Nadine Darwiche, Yehia Mechref

**Affiliations:** 1 Chemistry and Biochemistry Department, Texas Tech University, Lubbock, TX 79409, USA; mona.goli@ttu.edu (M.G.); vishal.sandilya@ttu.edu (V.S.); 2 Department of Biochemistry and Molecular Genetics, American University of Beirut, Beirut 1107 2020, Lebanon; bkg02@mail.aub.edu (B.G.); fkobaissy@msm.edu (F.K.); nd03@aub.edu.lb (N.D.); 3 Department of Experimental Pathology, Immunology and Microbiology, American University of Beirut, Beirut 1107 2020, Lebanon; he21@aub.edu.lb; 4 Center for Neurotrauma, Multiomics & Biomarkers, Department of Neurobiology, Morehouse School of Medicine, Atlanta, GA 30310, USA

**Keywords:** proteomics, adult T-cell Leukemia/Lymphoma, T-cell Acute Lymphoblastic Leukemia, ST1926, LC–MS/MS

## Abstract

T-cell malignancies represent a group of complex cancers arising from T cells and include aggressive subtypes such as Adult T-cell Leukemia/Lymphoma (ATL) and T-cell Acute Lymphoblastic Leukemia (T-ALL). Patients with these aggressive subtypes still represent an unmet medical condition. The synthetic adamantyl retinoid ST1926, a potent DNA polymerase-α inhibitor, proved a promising potency in preclinical models of ATL and peripheral T-cell lymphoma. Using advanced liquid chromatography–mass spectrometry (LC–MS/MS) techniques, we explored the effects of ST1926 on global protein expression in ATL (HuT-102) and T-ALL (MOLT-4) cells. We demonstrate that ST1926 triggers differentiation and apoptosis in malignant T-cells while halting tumor progression. Evidence at the proteomics level reveals the impact of ST1926 on crucial DNA replication enzymes and cell cycle regulation, highlighting its potential to reduce leukemogenesis and promote apoptosis. Our findings underscore the potential of ST1926 as an innovative therapeutic approach to address these aggressive T-cell malignancies, providing valuable insights into developing new targeted therapies and improving the outcomes and prognosis of patients with these challenging diseases.

## 1. Introduction

T-cell malignancies represent a diverse group of disease subtypes related to abnormal malignant T cells produced at different stages of their clonal evolution. These malignancies include aggressive subtypes such as Adult T-cell Leukemia/Lymphoma (ATL) and T-cell Acute Lymphoblastic Leukemia (T-ALL). ATL is a rare and aggressive hematological malignancy secondary to the infection by the human T-cell lymphotropic virus type 1 (HTLV-1) [1]. This virus primarily affects CD4+ T-cells, leading to their uncontrolled proliferation and subsequent development into malignant cells [2]. The pathogenesis of ATL involves complex interactions between the viral oncogenes, particularly Tax and HBZ, with key host cellular pathways, leading to the transformation of infected T-cells and the suppression of the host immune response [3,4]. ATL is characterized by a range of clinical manifestations, including lymphadenopathy, hepatosplenomegaly, skin lesions, and hypercalcemia, which can lead to various complications [5]. The standard treatment regimens include chemotherapy and antiviral agents, often resulting in low response rates, highlighting the need for more effective therapies [6]. This urges novel therapeutic approaches to improve outcomes for patients with ATL.

T-ALL arises from genetic mutations and the abnormal proliferation of immature progenitors. Risk-based therapies have significantly improved the survival of T-ALL patients; however, mortality remains high due to relapse, therapy resistance, or treatment-related complications [7]. Relapsed T-ALL has poor outcomes, with event-free and overall survival rates below 25%. The primary goal in relapsed T-ALL patients is to achieve remission, followed by allogeneic hematopoietic cell transplantation for a complete response [8].

Retinoids are a class of chemical compounds known to play a critical role in regulating cell growth, differentiation, and apoptosis, making them promising in cancer treatment [9,10]. They include both natural and synthetic analogs that share structural or functional similarities with vitamin A [11]. Retinoids have shown promising potency in ATL by inducing differentiation and apoptosis in malignant T-cells, inhibiting tumor progression [12]. Despite the potential of retinoids in cancer therapy, their use is often limited by toxic side effects, decreased stability, and the development of resistance in patients. To address these challenges, synthetic retinoids have been developed as alternatives, namely the adamantyl retinoids, including ST1926 [13,14].

Synthetic retinoids (STs) have been investigated in terms of their ability to treat a variety of cancers since the late 20th century. Furthermore, in the past decade, STs have displayed promising results by themselves or in synergy with other treatments for the treatment of breast cancer, melanoma, and colorectal cancer [15]. Peretinoin, a binder of Retinoid X Receptor (RXR) and Retinoid Acid Receptor (RAR), has displayed promising results in the treatment of liver cancer [16]. By contrast, WYC-209, which selectively targets RAR, has shown great efficacy in the treatment of melanoma and gastric cancer [17,18]. Lastly, ST1926, another synthetic retinoid, has demonstrated consistent pharmacokinetic properties and bioavailability while producing minimal side effects when used against solid tumors. Additionally, ST1926 has been shown to exhibit anti-cancer properties against colorectal, breast, and prostate cancers through its ability to bind to RARs [19,20].

We previously examined the efficacy of ST1926 in ATL models in vitro and in vivo [21]. We reported that pharmacologically achievable levels of ST1926 induced cell death in both all-trans retinoic acid (ATRA)-resistant HTLV–1-positive and -negative malignant T-cell lines, as well as in primary ATL cells, with no effect on normal lymphocytes. Importantly, ST1926 reduced tumor size and extended the lifespan of ATL murine models [21]. On the molecular level, ST1926 reduced the expression of the viral oncoprotein Tax and induced apoptosis both in vitro and in vivo.

Using the advanced liquid chromatography–tandem mass spectrometry (LC–MS/MS) technique, we explored the impact of ST1926 on protein expression in both ATL and T-ALL malignant T-cells. Our findings offer valuable insights into the mechanisms underlying ST1926 antitumor efficacy and in shaping innovative treatments for aggressive T-cell malignancies.

## 2. Results and Data Analysis

### 2.1. Protein Identification and Quantification

The lists of quantified proteins in ATL (HuT-102) and T-ALL (MOLT-4) malignant T cells after treatment with ST1926 at different time points (2, 12, and 24 h), along with the protein IDs, protein names, and gene IDs, are provided in Table S1. Log2 normalization was utilized in Perseus. Following Perseus quantitation, R (version 4.4.1) and Python (version 3.12.1) scripts were employed for statistical analysis, gene ontology analysis, and figure generation. In total, 2291 (Table S4) and 2099 proteins (Table S5) were quantified in HuT-102 and MOLT-4 cells, respectively. A total of 1870 common proteins were identified between the two cell lines, with 421 proteins unique to HuT-102 cells and 229 proteins unique to MOLT-4 cells (Venn diagram in Figure S2).

### 2.2. Principal Component Analysis of Protein Expression in HuT-102 and MOLT-4 Cells

**Figure 1:**
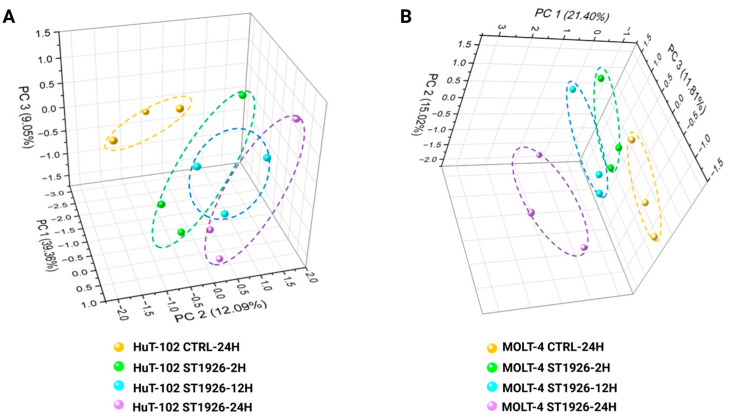
Unsupervised Principal Component Analysis (PCA) of the whole proteome expressed in (**A**) Adult T-cell Leukemia/Lymphoma (HuT-102) and (**B**) T-cell Acute Lymphoblastic Leukemia (MOLT-4) malignant T cells after treatment with ST1926 at different time points (2, 12, and 24 h).

Unsupervised principal component analysis was performed on the overall log2-transformed data, and the first three principal components, ranked based on their variance explained, were displayed using a 3-dimensional scatter plot, as represented in Figure 1. The PCA plots display significant separation between the control and ST1926 treatment groups for both HuT-102 and MOLT-4 cells, indicating significant changes in the proteome brought upon by ST1926 treatment, with the most considerable separation being between the control and 24 h post-ST1926 treatment for both HuT-102 and MOLT-4 cells.

### 2.3. Analysis of Differentially Expressed Proteins via Volcano Plots

**Figure 2:**
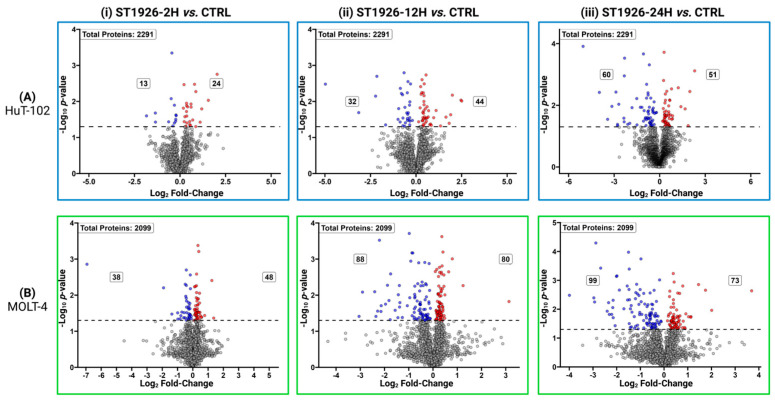
Volcano plots showing the distribution of quantified proteins in (**A**) Adult T-cell Leukemia/Lymphoma (HuT-102) and (**B**) T-cell Acute Lymphoblastic Leukemia (MOLT-4) malignant T cells at (i) 2 h, (ii) 12 h, and (iii) 24 h treatment with ST1926 according to the *p*-value. Significant levels are indicated with the horizontal lines (at *p*-value < 0.05) and color-coded dots (red represents the up-regulated proteins; blue represents the down-regulated proteins (*p*-value < 0.05; gray represents the proteins without significant expressions).

The volcano plots in Figure 2 represent the significantly altered proteins (*p*-value < 0.05) in HuT-102 (Figure 2A) and MOLT-4 cells (Figure 2B) at (i) 2, (ii) 12, and (iii) 24 h post-ST1926 treatment. The list of significant proteins, along with their increased or decreased abundance status for each comparison for HuT-102 and MOLT-4 cells, are provided in Tables S2 and S3, respectively, along with their protein IDs, protein names, gene IDs, *p*-values, and FCs. For HuT-102 cells, at 24-h post ST1926-treatment, RRBP1 (*p*-value = 5.6 × 10^−5^, fold-change = 0.07), and TIMP1 (*p*-value = 1 × 10^−4^, fold-change = 0.03) were the two most significantly decreased proteins while HIBADH (*p*-value = 7.7 × 10^−4^, fold-change = 4.93) and ITPA (*p*-value = 2 × 10^−4^, fold-change = 1.22) were the two most significantly increased. In contrast, for MOLT-4 cells, UBE2C (*p*-value = 4 × 10^−4^, fold-change = 0.15) and EMD (*p*-value = 5 × 10^−5^, fold-change = 0.14) were the two most significantly decreased proteins while SOD2 (*p*-value = 1 × 10^−3^, fold-change = 1.34) and ATP5F1B (*p*-value = 6 × 10^−4^, fold-change = 1.3) were the two most significantly increased.

### 2.4. Protein Expression Analysis Using Hierarchically Clustered Heatmaps

For further protein expression analysis, hierarchically clustered heatmaps were constructed for similarly altered proteins. Thus, proteins that are closer together have similar expressions. Figures S3 and S4 demonstrate differences in significant protein expressions among pairwise comparisons in HuT-102 and MOLT-4 cells after treatment with ST1926 at different time points (2, 12, and 24 h) using hierarchical clustering heatmaps.

**Figure 3:**
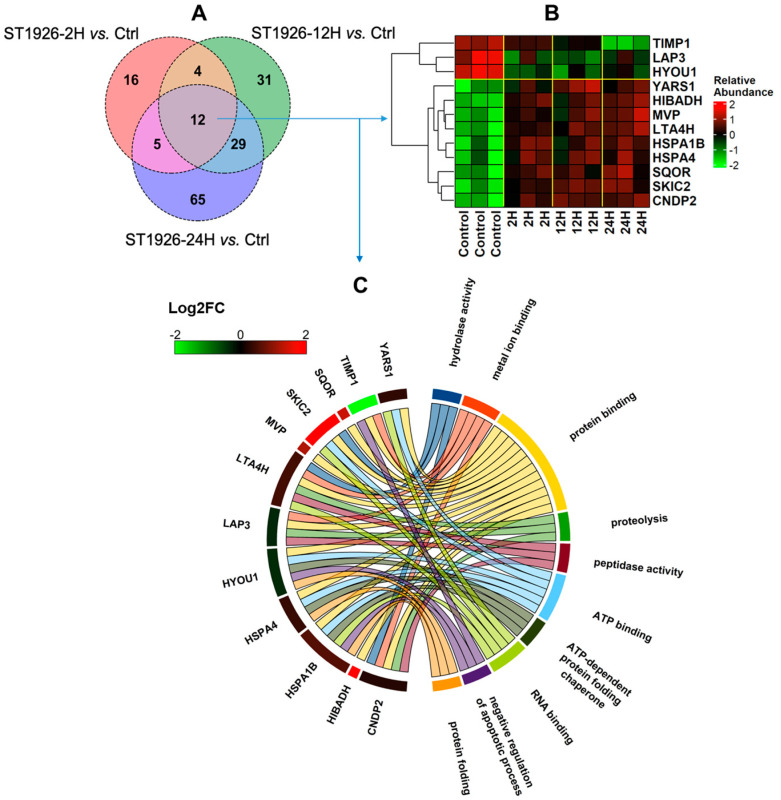
(**A**) Venn diagram comparing the proteins with significant alterations in Adult T-cell Leukemia/Lymphoma (HuT-102) upon treatment with ST1926 at different time points (2, 12, and 24 h); (**B**) Hierarchical heatmap clustering representing the expression of 12 common proteins among different timepoints treatment with ST1926; relative abundance was calculated via the z-score, with values below the mean being negative and values above the mean being positive; (**C**) Biological process annotation of proteins commonly altered across each time point in HuT-102 cells.

**Figure 4:**
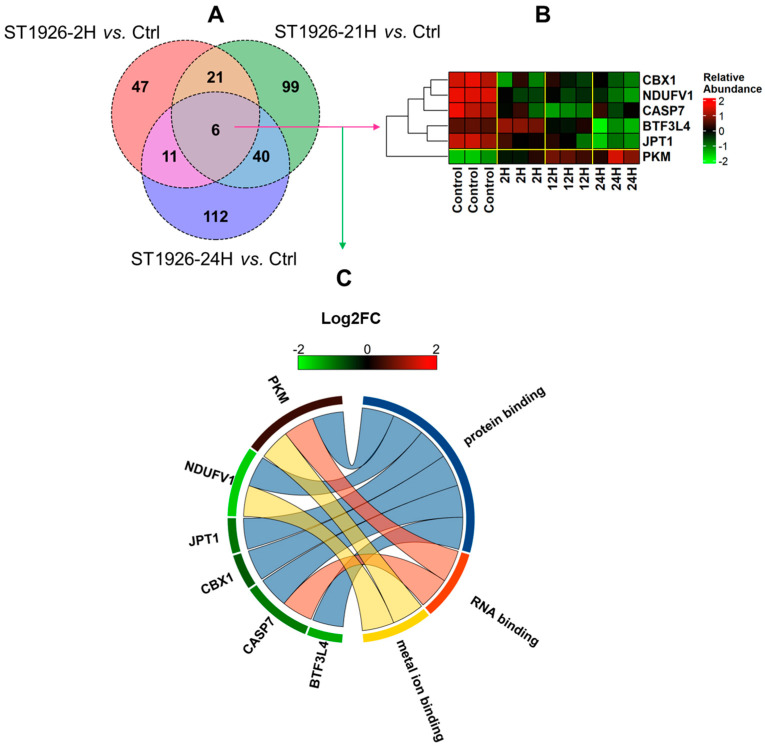
(**A**) Venn diagram comparing the proteins with significant alterations in T-cell Acute Lymphoblastic Leukemia cells (MOLT-4) upon treatment with ST1926 at different time points (2, 12, and 24 h); (**B**) Hierarchical heatmap clustering representing the expression of 6 common proteins among different treatment timepoints with ST1926; relative abundance was calculated via z-score, with values below the mean being negative and values above the mean being positive; (**C**) Biological process annotation of proteins commonly altered across each time point in HuT-102 cells.

Proteins commonly altered between different time points were identified, and the results are displayed in the Venn diagram in Figure 3A and Figure 4A. For HuT-102, 12 proteins were differentially expressed across each time point. Metalloproteinase inhibitor 1 (TIMP1), cytosol aminopeptidase (LAP3), and hypoxia upregulated protein 1 (HYOU1) were consistently decreased, while tyrosine-tRNA ligase, cytoplasmic (YARS1), 3-hydroxyisobutyrate dehydrogenase, mitochondrial (HIBADH), major vault protein (MVP), leukotriene A-4 hydrolase (LTA4H), heat shock 70 kDa protein 1B (HSPA1B), heat shock 70 kDa protein 4 (HSPA4), sulfide: quinone oxidoreductase, mitochondrial (SQOR), superkiller complex protein 2 (SKIC2), and cytosolic non-specific dipeptidase (CNDP2) were increased in post-treatment versus control. Figure 3A displays the Venn diagram comparing significantly altered proteins across each timepoint, while Figure 3B,C represents the heatmap and the Gene ontology (GO) term annotations (Circos plot) for the 12 commonly differentially expressed proteins, respectively. GO term annotation for the common proteins across different treatment time points revealed central involvement in protein binding, ATP binding, hydrolase activity, and nucleotide binding. In this investigation, the clusterProfiler [22] package was used along with the genome-wide annotation for humans for the GO analysis.

In contrast, 6 proteins were significantly altered across each time point for MOLT-4, as represented by the Venn diagram in Figure 4A. The expression of Chromobox protein homolog 1 (CBX1), NADH dehydrogenase [ubiquinone] flavoprotein 1, mitochondrial (NDUFV1), caspase-7 (CASP7), and Jupiter microtubule-associated homolog 1 (JPT1) was consistently decreased, pyruvate kinase PKM (PKM) was consistently increased, and transcription factor BTF3 homolog 4 (BTF3L4) was initially increased at the 2-h mark but then was observed to be decreased (Figure 4B). The GO term annotation for the six proteins displayed involvement in protein, RNA, and metal binding (Figure 4C).

Lastly, a Venn diagram was constructed to identify commonly altered proteins between HuT-102 and MOLT-4 cells at 24 h post-treatment. A total of 13 proteins were commonly differently expressed in the two groups; 98 were uniquely significant in HuT-102, while 156 were unique to MOLT-4. The majority of these 13 proteins displayed a similar trend of regulation (increase vs. decrease in abundance) for both HuT-102 and MOLT-4, except heterogeneous nuclear ribonucleoprotein A3 (HNRNPA3), which was increased in HuT-102 and decreased in MOLT-4 cells. The bar plots (made in R v4.4.2) for the 13 common proteins, along with the Venn diagram for the comparison, are presented in Figure S5.

### 2.5. Hallmark Gene Set Enrichment, Pathway Analysis, and Protein–Protein Interaction Analyses

In HuT-102 cells, Hallmark gene set enrichment analysis revealed significant alterations in pathways such as mammalian target of rapamycin complex 1 pathway (mTORC1) signaling, myelocytoma (MYC) Targets V1, early region 2 binding factor (E2F) targets, adipogenesis, oxidative phosphorylation, and G2-M checkpoint regulation (Figure S6A). MOLT-4 cells exhibited similar alterations in oxidative phosphorylation, G2-M checkpoint regulations, and MYC Targets V1, in addition to disruptions in other cancer-related processes such as protein secretion and DNA repair (Figure S6B).

GO enrichment analysis indicated a substantial disruption in RNA binding functions in both cell lines at 24 h post-ST1926 treatment (Supplementary Figures S7C and S8C). Furthermore, analysis of cellular components revealed significant alterations in proteins associated with intracellular membrane-bounded organelles, particularly the nucleus and mitochondria (Supplementary Figure S7B and S8B). Notably, the largest difference was observed in the biological processes of significantly altered proteins in HuT-102 and MOLT4 cells, with the majority of the differentially expressed proteins for HuT-102 being involved in the regulation of ubiquitin protein ligase activity and ribosomal small subunit biogenesis (Supplementary Figure S7A). In contrast, the majority of the differentially expressed proteins in MOLT-4 were involved in gene expression and ATP synthesis-related pathways (Supplementary Figure S8A).

In HuT-102 cells, IPA revealed the downregulation of critical pathways related to DNA synthesis, tumor cell line invasion, and leukemia cell line proliferation (Figure S9). The inhibition of DNA synthesis was particularly evident, as crucial enzymes such as ubiquitin-conjugating enzyme E2C (UBE2C), ubiquitin-conjugating enzyme E2S (UBE2S), S-phase kinase-associated protein (SKP), cyclin-dependent kinase 2 (CDK2), and DNA replication complex GINS protein PSF3 (GINS3) were markedly decreased in expression. Additionally, the downregulation of receptor tyrosine kinase erbB2 (ERBB2, also known as HER2) was inferred from the decreased expression of several interacting proteins, including thymidylate synthase (TYMS), transcription factor JunB (JUNB), condensing complex subunit 1 (NCAPD2), and ribonucleoside-diphosphate reductase large subunit (RRM1).

In MOLT-4 cells, pathways related to mitotic metaphase and anaphase, processing of capped intron-containing pre-mRNA, and RNA polymerase II transcription were notably inhibited (Figure S10). In contrast, the Sirtuin signaling pathway was significantly activated. The downregulation of ERBB2 was similarly inferred, based on the decreased protein levels of DNA polymerase alpha subunit B (POLA2), DNA topoisomerase 2-alpha (TOP2A), and condensin complex subunit 3 (NCAPG), coupled with the increase in abundance of nuclear pore membrane glycoprotein 210 (NUP210) and transmembrane emp24 domain-containing (TMED9).

**Figure 5:**
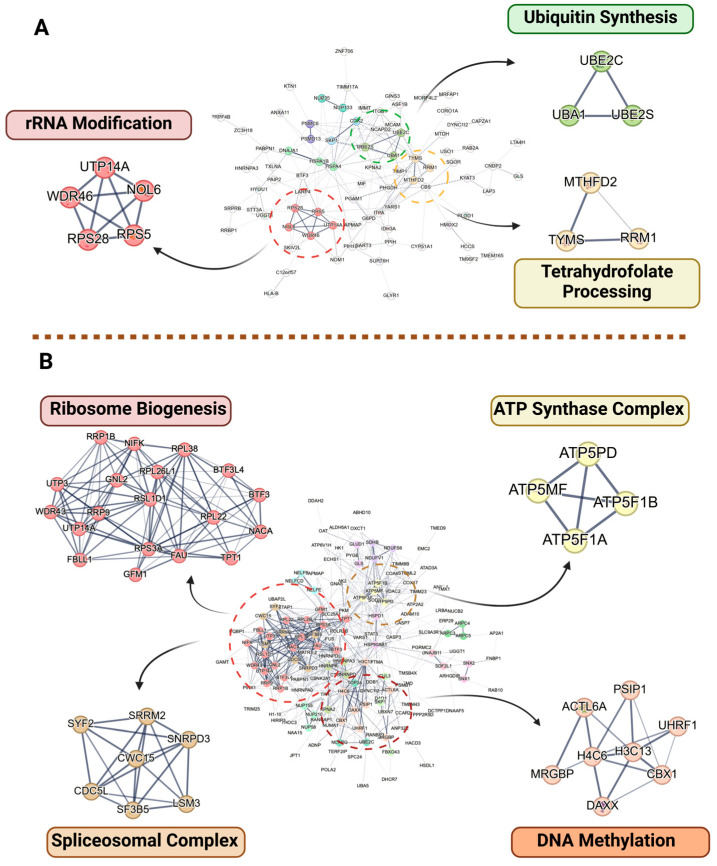
Protein–protein interaction (PPI) network for differentially expressed protein in (**A**) Adult T-cell Leukemia/Lymphoma (HuT-102) and (**B**) T-cell Acute Lymphoblastic Leukemia T-cells (MOLT-4) after 24 h of treatment with ST1926. These PPI networks illustrate the interactions among proteins (nodes), where connections (edges) represent co-expression, co-localization, or involvement in shared biological pathways. Additionally, the spatial proximity of two proteins within a PPI network reflects a strong correlation in their expression profiles and functional associations.

In the PPI network for HuT-102 cells, three distinct clusters were readily observed: one comprising proteins in ubiquitin synthesis, one in rRNA modification, and the other focused on tetrahydrofolate processing (Figure 5A). In MOLT-4 cells, four protein clusters were identified and associated explicitly with ribosome biogenesis, the ATP synthase complex, the spliceosomal complex, and DNA methylation (Figure 5B).

## 3. Discussion

Cancer cells are known to exploit cellular translational machinery to promote cell proliferation and cancer metastasis. Our analysis revealed a common disruption of protein translation and ribosome biogenesis in both ST1926-treated HuT-102 and MOLT4 cells, suggesting an overall decrease in cell proliferation and metastatic potential after ST1926 treatment.

The expression of HNRNPA3, a multifunctional RNA-binding protein, appears to be significantly altered in treated cells [23]. HNRNPA3 processes capped intron-containing pre-mRNA as a part of the eukaryotic spliceosome complex. Additionally, HNRNPA3 is involved in the cytoplasmic trafficking of RNA and is known to interact with the Tat protein of HIV-1, another retrovirus, to facilitate viral genome replication. We hypothesize that a similar interaction may occur in HuT-102 cells, which could explain the observed expression changes following ST1926 treatment.

For ST1926-treated HuT-102 cells, the protein–protein interaction analysis (Figure 5A) revealed a cluster of proteins involved in either translation or ribosome biogenesis via rRNA processing. This cluster includes ribosomal protein S5 and S28 (RPS5 and RPS28), La-Related Protein 4 (LARP4), a nucleolar protein with MIF4G domain 1 (NOM1), WD Repeat Domain 46 (WDR46), Nucleolar Protein 6 (NOL6), and Poly(A)-Binding Protein Nuclear 1 (PABPN1). RPS5 and RPS28 are both constituents of the small subunit (40S) of the eukaryotic ribosome and were both found to be decreased in abundance after treatment with ST1926. Furthermore, RPS27A, another member of the small subunit, is known to promote the proliferation of leukemic cells [24] and was initially reduced at 12 but not at 24 h. WDR46—a nucleolar protein—is another protein with decreased expression that is predicted to be a part of the small ribosome subunit processome [25].

Similarly, ST1926-treated MOLT4 cells exhibited disruptions in translation and ribosome biogenesis through reduction in several small ribosomal subunit components, including small ribosomal subunit protein eS25 (RPS25), small ribosomal subunit protein eS1 (RPS3A), and small ribosomal subunit protein eS4, X isoform (RPS4X). The ribosomal L1 domain containing 1 (RSL1D1) protein, which plays a role in ribosome biogenesis [26,27] and functions as a transcriptional cofactor, was also decreased in the ST1926-treated MOLT4 cells.

PABPN1 and CDC123 are translational proteins with ubiquitous expression in the lymph nodes [28]. PABPN1 binds to pre-mRNA’s nascent poly (A) tails and is required for efficient tail polymerization [29,30,31]. A decrease in PABPN1 in ST1926-treated HuT-102 cells suggests destabilization of the mRNA maturation and transport process. CDC123, thought to be a member of the eukaryotic translation initiation factor 2 complex, is responsible for the positive regulation of translation initiation [32]. The concurrent attenuation of these proteins upon ST1926 treatment implies a destabilization of the protein synthesis machinery associated with the small ribosomal subunit and mRNA. The decrease in protein synthesis is further supported by the downregulation of JUNB, which plays a role in the positive regulation of transcription via RNA polymerase II and is also known to be involved in angiogenesis in various cancers, including multiple myeloma [33,34], breast cancer [35], and renal cell carcinoma [36].

The suppression of cell proliferation in MOLT-4 cells 24 h after ST1926 treatment was evident by the downregulation of UBE2C and SKP1, which are involved in cell-cycle progression [37,38,39]. TOP2A is highly expressed during DNA replication and assists in relieving torsional strain occurring during DNA replication and translation [40]. Similarly, POLA2, a part of the DNA Polymerase–primase complex, is heavily involved in DNA replication and the synthesis of RNA primers [41]. Overexpression of TOP2A and POLA2 has been strongly associated with enhanced cancer cell proliferation, and the observed downregulation of these proteins following ST1926 treatment indicates a potential reduction in cellular proliferation activity [42,43,44,45]. Furthermore, it has been shown that ST1926 treatment leads to downregulation of POL2A in glioblastoma cell lines [46]. RSL1D1 inhibits cellular senescence—a state of cell cycle arrest for aged cells—in various cancers, such as colorectal cancer [27,47,48]. The observed reduction in RSL1D1 abundance in treated MOLT4 cells suggests increased cell-cycle arrest, mirroring the effects observed in HuT-102 cells. Furthermore, the downregulation of nucleolar GTP-binding protein 2 (GNL2/NGP1), another critical factor in ribosome biogenesis, reinforces the observed reduction in ribosome biogenesis. Given GNL2’s role in promoting the G1 to S phase transition [49] and its overexpression in various cancers, this downregulation strongly implies a cell cycle arrest before the G2-M transition.

Ubiquitin-like with PHD and ring finger domains 1 (UHRF1), belonging to a subfamily of RING-finger type E3 ubiquitin ligases, is responsible for the recruitment of DNA (cytosine-5)-methyltransferase 1 (DNMT1) for methylation of DNA [50,51]. UHRF1 is markedly overexpressed in acute myeloid leukemia (AML) cells, with elevated levels strongly correlating with poor prognosis [52,53]. This overexpression of UHRF1 is essential for the aberrant self-renewal of leukemia-initiating cells, which drives AML cell proliferation [52,53]. Following ST1926 treatment, the observed downregulation of UHRF1 in MOLT4 cells further indicates a potential suppression of leukemogenesis via cell cycle arrest before the M phase.

A reduction in DNA synthesis was uniquely observed in the ST1926-treated HuT-102 cells compared to the control, as indicated by the downregulation of several vital proteins with the gene IDs of GINS3, CDK2, UBE2S, UBE2C, and SKP1. In eukaryotic cells, DNA replication is a tightly regulated process that requires the coordination of multiple essential factors, collectively known as the replisome [54]. GINS3 is a member of the GINS complex, which plays a critical role in establishing DNA replication forks and facilitating the progression of the replisome [55,56,57]. Cyclin-dependent kinases (CDKs) are another crucial component of the replication complex. The accumulation of cyclin E at the end of the G1 phase activates CDK2, promoting the G1-S transition. In contrast, the accumulation of cyclin A promotes the transition from the S phase to the M phase [58,59].

Polyubiquitination, followed by proteasomal degradation, drives the cell cycle and is regulated by SKP1-CUL1-F-box proteins and the anaphase-promoting complex/cyclosome (APC/C) [60,61]. UBE2C and UBE2S are responsible for elongating the polyubiquitin chain; thus, their downregulation and SKP1 suggest another instance of cell cycle arrest at the S phase [37,38,39]. Additionally, TYMS, an enzyme vital for DNA replication, was also observed to decrease at 12- and 24-h post-ST1926 treatment. TYMS catalyzes the methylation of deoxyuridylate to deoxythymidylate, ensuring the maintenance of deoxythymidine monophosphate (dTMP) levels required for DNA replication and repair; an increase in TYMS expression is directly correlated with genomic instability [62,63].

Mitochondrial function is crucial for ATP production through the tricarboxylic acid (TCA) cycle and oxidative phosphorylation, and mitochondrial dysfunction has been implicated in various cancers, including leukemia and lymphoma [64,65]. The hierarchically clustered heatmap and the PPI plot for significant proteins revealed a cluster of mitochondrial proteins that were increased at 24 h post-ST1926 treatment in MOLT-4 cells. Mitochondrial superoxide dismutase (SOD2), responsible for scavenging superoxide radicals in the mitochondria, also acts as a tumor suppressor by mediating the superoxide-to-peroxide ratio [66]. Given that superoxide radicals are known to accumulate to sub-lethal levels during mitosis, the increase in expression of SOD2 may reduce cell proliferation by scavenging these radicals. Similarly, succinate dehydrogenase complex iron sulfur subunit B (SDHB), another mitochondrial enzyme with tumor suppressor properties, showed increased levels after ST1926 treatment. SDHB is one of the four members of the succinate dehydrogenase (SDH) complex, which catalyzes succinate into fumarate during the TCA cycle [67,68]. Succinate is known to promote oncogenesis and tumor progression [69], with mutations in SDH being commonly linked to carcinoma [70,71,72]. The increased abundance of SOD2 and SDHB suggests an enhanced tumor-suppressing effect of ST1926.

Moreover, a significant increase in expression of several ATP synthase subunits was observed 24 h post-ST1926 treatment. ATP synthase, an integral component of the electron transport chain located in the inner mitochondrial membrane, catalyzes the conversion of ADP into ATP. Sarcoplasmic/endoplasmic reticulum calcium ATPase 2 (ATP2A2), ATP synthase subunit alpha, mitochondrial (ATP5F1A, also known as ATP5A1), ATP synthase subunit beta, mitochondrial (ATP5F1B), ATP synthase subunit f, mitochondrial (ATP5MF), ATP synthase membrane subunit K, mitochondrial (ATP5MK), ATP synthase subunit d, mitochondrial (ATP5PD), and ATP synthase-coupling factor 6, mitochondrial (ATP5PF) exhibited notable increases in protein levels across various time points. Loss of these subunits has been implicated in mitochondrial reprogramming, which is associated with cancer therapy resistance and typically correlates with a poor prognosis. Moreover, ATP5A1, which was significantly increased upon ST1926 treatment, is also known to have a pro-apoptotic effect.

Voltage-dependent anion channel 2 (VDAC2), an ion-gated channel situated in the outer mitochondrial membrane, was also increased upon ST1926 treatment. VDAC2 is critical for releasing cytochrome C and other pro-apoptotic factors, facilitating apoptotic signaling pathways [73]. Conversely, glutaminase (GLS), primarily localized in the mitochondria, catalyzes the conversion of glutamine to glutamate—a reaction essential for the rapid proliferation of cancer cells—[74,75] and was found to be decreased. This downregulation suggests a potential decrease in the metabolic energy supply available to cancer cells. Similar downregulation of GLS was also observed in HuT-102-treated cells.

Although there were similarities in pathway alterations in ST1926-treated ATL (HuT-102) and T-ALL (MOLT-4) cells, there were also notable differences between the two types of leukemic cells. These variations can be attributed to the cellular origin, genetic background, and different oncogenic drivers. In fact, HuT-102 cells arise from T-cells infected with the HTLV-1. As such, ATL cells often carry specific mutations or genetic alterations related to the virus, such as the expression of viral oncoproteins like Tax [4]. However, MOLT-4 cells arise from malignant T-cell progenitors and often involve mutations in genes such as NOTCH1 (a key oncogenic driver that activates downstream pathways like PI3K/Akt, MAPK, and JAK/STAT), CDKN2A, PTEN, and others [76]. Furthermore, the two types of leukemic cells are characterized by different signaling pathways. HuT-102 cells often show activated NF-κB signaling due to HTLV-1 infection, which results in the production of pro-survival factors, anti-apoptotic proteins, and cytokines that sustain tumor cell survival and proliferation [77]. However, MOLT-4 cells exhibit a primary alteration in the activation of NOTCH1 signaling. Mutations in NOTCH1 lead to abnormal transcriptional activation of target genes, influencing cell cycle regulation and promoting the transition from immature thymocytes to malignant T-cells. There are often alterations in tumor suppressor pathways, such as the loss of PTEN, which can further drive aberrant cell growth and survival in T-ALL.

ST1926 has previously been shown to promote an anti-leukemic effect in AML [78] and glioblastoma [46] cell lines through the perturbation of cellular replication machinery, resulting in cell-cycle arrest and the induction of apoptotic factors. Our results align with the previous findings, demonstrating that ST1926 has similar effects of cell-cycle arrest and an overall increase in apoptosis in previously unstudied T-ALL and ATL cell lines.

## 4. Materials and Methods

### 4.1. Materials and Reagents

The HTLV-1-transformed CD4+ T-cell line (HuT-102) and the human T-ALL cell line (MOLT-4) were purchased from the American Tissue Culture Collection (ATCC, Manassas, VA, USA). RPM-I medium (Lonza, Walkersville, MD, USA) and fetal bovine serum for cell lines were purchased from GIBCO BRL, Gaithersburg, MD, USA) ST1926 was obtained from Biogem Institute (Ariano Irpino, Italy) and MedChemExpress (Monmouth Junction, NJ, USA). ST1926 was prepared as stock solutions in dimethylsulfoxide (DMSO) at 1 × 10^−2^ M in amber tubes and stored at −80 °C.

High-performance liquid chromatography (HPLC)-grade water, acetonitrile (ACN), and formic acid (FA) were provided by Fisher Scientific (Fair Lawn, NJ, USA). The ammonium bicarbonate (ABC), sodium deoxycholate (SDC), dithiothreitol (DTT), and iodoacetamide (IAA) were supplied by Sigma-Aldrich (St. Louis, MO, USA). The mass-spectrometry-grade Trypsin/Lys-C Mix was purchased from Promega (Madison, WI, USA).

### 4.2. Cell Culture and Drug Administration

HuT-102 and MOLT-4 cells were seeded at a density of 3 × 10^5^ cells/mL. Cell culture media was changed 24 h post-cell seeding. Afterward, cells were treated with 1 µM of ST1926 for the indicated time points (2, 12, and 24 h). Control cells were treated with DMSO and were collected at the maximal hour of treatment, i.e., just for 24 h. The final concentrations of DMSO in the control cells never exceeded 0.1%, a level that had no effect on the growth of any of the tested ATL or malignant T cells up to 24 h (Figure S1). Additionally, it has previously been shown that 0.1% DMSO has no effect on the growth of any ATL or malignant T cells up to 72 h [21]. We selected an ST1926 concentration of 1 μM, since it results in more than 90% of growth inhibition at 48 h in all tested cells, with no effect on resting or activated human peripheral blood mononuclear cells [21]. The selected times of treatment for ATL and MOLT-4 cells were less than 48 h, i.e., the growth inhibition of cells was much lower at earlier time points (2, 12, 24 h) as published [21]. Importantly, there were no observed toxicity signs in resting or activated human peripheral blood mononuclear cells that were treated with 10-fold higher concentrations of ST1926 (10 µM) [21]. Experiments were repeated independently three times, and each time point was reproduced in three biological replicates on the same passage of tested cells. Cells were harvested at the indicated time points without the use of trypsin, and cell pellets were washed twice with 1× PBS and stored at −80 °C for later use for LC–MS/MS proteomics analysis.

### 4.3. Cell Lysis and Protein Extraction

The frozen cells were thawed at room temperature, then combined with a 5% SDC solution along with 400 μm molecular biology-grade zirconium beads (BMBZ 400-250-36, OPS Diagnostics, LLC, Lebanon, NJ, USA) in a 2 mL microtube. The 5% SDC solution was introduced to facilitate protein extraction using a bead beater (Beadbug microtube homogenizer, Benchmark Scientific, Edison, NJ, USA) at 4 °C. The bead beater was operated at 4000 revolutions/min for 30 seconds (s), followed by a 30 s pause for cooling, and this cycle was repeated five times. Subsequently, the cell lysate was sonicated in an ice–water bath for 30 min to enhance protein dissolution. Following this, the samples underwent centrifugation at 21,000× *g* for 10 min, and the resulting supernatant was collected and then diluted twentyfold with 50 mM ABC buffer to mitigate interference from the 5% SDC during proteomics analysis. Notably, this step was conducted in a time-controlled manner, with each sample being exposed to this procedure for the same number of total cycles and similar durations.

### 4.4. Protein Digestion

Before conducting tryptic digestion, the protein concentration of the diluted lysed cell samples was determined using the micro-BCA protein assay, following the manufacturer’s instructions (Thermo Scientific/Pierce, Rockford, IL, USA). Subsequently, a 15 μg aliquot of extracted proteins from each HTLV-1 cell sample was subjected to reduction, alkylation, and tryptic digestion. The proteins were thermally denatured at 80 °C for 30 min. The reduction of proteins involved adding a 1.25 μL aliquot of 200 mM DTT solution (prepared in 50 mM ABC buffer) and incubating at 60 °C for 45 min. The reduced proteins were then alkylated by adding a 5 μL aliquot of the IAA solution (prepared in 50 mM ABC buffer) and incubated at 37.5 °C in the dark for 45 min. A second 1.25 μL aliquot of the DTT solution was added to the samples and incubated at 37.5 °C for 30 min to quench the excessive IAA. Following this, a 0.6 μg aliquot of Trypsin/Lys-C Mix was added to the reduced and alkylated proteins (enzyme/substrate ratio of 1:25 *w*/*w*) and incubated at 37.5 °C for 18 h. After incubation, FA (a final concentration of 0.5% *v*/*v*) was added to the samples to quench the enzymatic reaction and precipitate the SDC detergent used in the cell lysis step. The samples were thoroughly mixed, and the supernatant was collected by centrifugation at 14,800 rpm for 10 min. The supernatants were speed-vacuum dried and resuspended in 15 μL of 2% ACN and 0.1% FA for a final concentration of 1 μg/μL before LC–MS/MS proteomics analysis.

### 4.5. LC–MS/MS Proteomics Analysis

The tryptic digest, containing 1 μg of proteome for each sample, was introduced into a 3000 Ultimate nano-LC system (Thermo Fisher Scientific, San Jose, CA, USA) connected to an LTQ Orbitrap Velos mass spectrometer (Thermo Fisher Scientific, San Jose, CA, USA) with a nano-ESI source. A single injection per biological replicate was performed. To eliminate potential salts, the samples underwent online purification utilizing a trap column (Acclaim PepMap100 C18 cartridge, 75 μm I.D. × 2 cm, 3 μm particle sizes, 100 A° pore sizes, Thermo Scientific, San Jose, CA, USA). The purified samples were then separated using an Acclaim PepMap100 C18 capillary column (75 μm I.D. × 15 cm, 2 μm particle sizes, 100 A° pore sizes, Thermo Fisher Scientific, San Jose, CA, USA), with the column temperature maintained at 29.5 °C. Mobile phase A consisted of 2% ACN in water with 0.1% FA, while mobile phase B was 100% ACN with 0.1% FA. Peptides were separated using a 120-min gradient at a 350 nL/min flow rate. The gradient of mobile phase B was programmed as follows: 0–10 min, 5% B; 10–65 min, 5–20% B; 65–90 min, 20–30% B; 90–110 min, 30–50% B; 110–111 min, 50–80% B; 111–115 min, 80% B; 115–116 min, 80–5% B; and 116–120 min, 5% B. The full MS resolution was set to 60,000, with the mass-to-charge (*m*/*z*) range from 400 to 2000. The data-dependent acquisition mode was utilized for two scan events. In the tandem mass spectrometry MS/MS scan, collision-induced dissociation (CID) was applied to the top 10 most intense ions in a full MS scan event with a normalized collision energy of 35%, Q-value of 0.25, and activation time of 10 ms. The dynamic exclusion parameters included a repeat count of 2, a repeat duration of 30 s, an exclusion list size of 200, and an exclusion duration of 90 s.

### 4.6. Protein Quantification and Data Analysis

The MaxQuant software (version 1.5.4.1, Matrix Science Inc., Boston, MA, USA) was utilized to analyze the LC–MS/MS raw data, with protein quantitation based on ion intensity, and the data searched against the Swiss-Prot human database containing 20,415 protein entries. Fixed modifications included the carbamidomethylation of cysteine, while variable modifications consisted of the oxidation of methionine and protein N-terminal acetylation. Peptides were searched with a precursor mass tolerance of 6 ppm and a fragment mass tolerance of 0.5 Da. The minimal peptide length was set to 7 amino acids, allowing for a maximum of two missed cleavages. Only proteins with more than two identified peptides were taken into consideration. The false discovery rate (FDR) for peptide and protein identification was set to 0.01, with unique peptides and minimal numbers of razors set to one. Additionally, the “matching-between-run” function was enabled. The label-free quantification (LFQ) approach, utilizing at least two ratio counts, was employed to compare and normalize protein intensities across runs.

**Figure 6:**
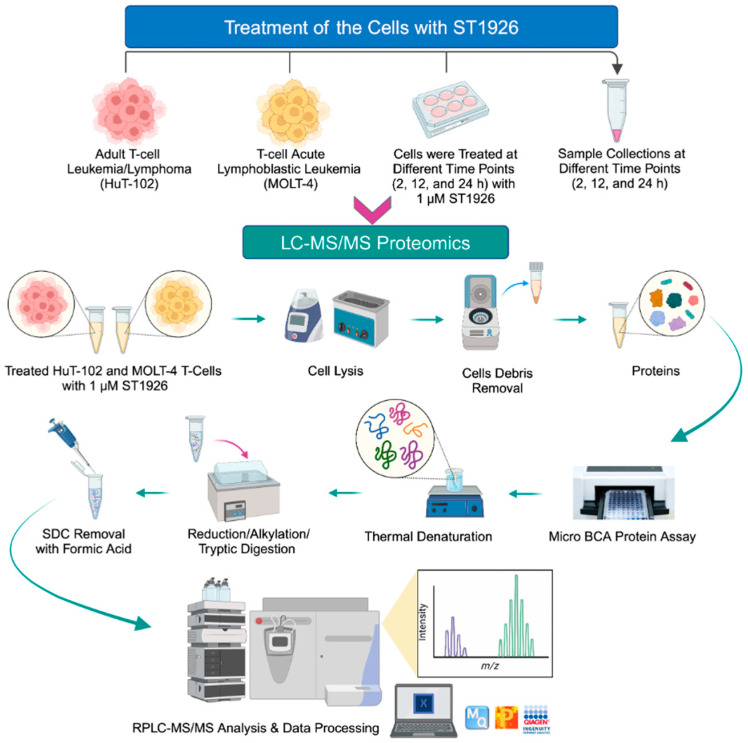
The workflow summarizing sample preparation and LC–MS/MS proteomics analysis.

Perseus version 1.5.5.0 (Max Planck Institute of Biochemistry, Munich, Germany), a supplementary software of MaxQuant, was employed afterward to provide the list of identified and quantified proteins. In Perseus, identified proteins from reversed sequences and contaminants were initially removed, leaving only proteins detected in over 70% of the run in at least one sample group. Furthermore, log2 normalization was employed in Perseus. Following quantification and normalization, statistical analysis was performed on the log2-transformed data to identify differentially expressed proteins. The control and treated cells were compared using Welch’s T-test for a pairwise comparison across each time point for both HuT-102 and MOLT-4 cells, with a *p*-value < 0.05 being defined as the significance threshold. The workflow summarizing sample preparation and LC–MS/MS proteomics analysis is presented in Figure 6.

Ingenuity Pathway Analysis (IPA, Qiagen, Hilden, Germany) was employed to identify significantly altered pathways and functions after ST1926 treatment. The Uniprot Swissprot accession IDs, along with their respective *p*-values and log2-fold change values for the significantly altered proteins (*p*-value < 0.05), were uploaded to IPA. A corrected *p*-value < 0.05 along with a |z-score| > 1 was used to identify significantly altered canonical pathways, diseases, and functions. No minimum number of proteins for enrichment was specified in IPA, with the |z-score| parameter and the corrected *p*-value serving as appropriate enrichment parameters. Gene ontology analyses (biological processes, molecular functions, and cellular components) for significantly altered proteins (*p*-value < 0.05) were performed in R via the clusterProfiler package along with the org.Hs.eg.db database [22]. Gene set enrichment analysis was performed using the Hallmark database constructed from the aggregation of various MSigDB (v7.5.1) package datasets in R [79]. A BH-corrected *p*-value < 0.05 was used as the cutoff criterion for all gene ontology analyses. The Uniprot-swissprot accession IDs for significantly altered proteins (*p*-value < 0.05) were uploaded to StringDB (https://string-db.org/, accessed on 20 October 2024) to identify protein–protein interactions. The entire STRING network, containing both functional and physical protein associations, was utilized and proteins were filtered to only retain those with at least one or more interactions with an interaction score greater than 0.4. Furthermore, only the interactions of the query proteins were analyzed, with no additional interactors added to the analysis.

The experimental workflow (Figure 6) was generated via BioRender (accessed on 14 April 2024). The volcano plots, circos plots, GO barplots, and protein abundance barplots were made using the ggplot2 and circlize packages in R (v 4.4.1) and biorender [80,81]. The Venn diagrams were constructed using the matplotlib package in Python (v3.12) [82]. The hierarchically clustered heatmaps (Figures S2 and S3) were made using Genesis [83]. String analysis figures were generated via the StringDB website and further modified in BioRender. Lastly, the unsupervised principal component analysis (Figure 1) was performed and plotted in OriginPro (OriginLab).

## 5. Conclusions

This study evaluated the efficacy of the adamantyl retinoid ST1926 for treating ATL (HuT-102) and T-ALL (MOLT-4) malignant T-cells. Building upon our previous findings demonstrating ST1926’s antitumor activity in glioblastoma and ATL, we now present comprehensive proteomic evidence for its efficacy in impeding leukemogenesis through G1/S phase cell cycle arrest induction and an increase in apoptosis.

In ST1926-treated MOLT-4 cells, reduced cell proliferation and increased cell cycle arrest were evident from the downregulation of crucial DNA replication enzymes, including TOP2A, POLA2, UHRF1, and GLS, alongside the increase in expression of SOD2 and SDHB. ST1926 similarly reduced TOP2A and POLA2 in glioblastoma cell lines. Although POLA2 and TOP2A were not quantified in HuT-102 cells, the decrease in cell proliferation was still evident from the downregulation of GINS3, CDK2, SKP1, HYOU1, UBE2C, and UBE2S. Additionally, disruption of the small ribosomal subunit components and associated proteins appears to be a central mechanism through which ST1926 exerts its effects. The concurrent decrease in UHRF1 and increase in pro-apoptotic factors upon ST1926 treatment further reinforces the compound’s potential in suppressing leukemogenesis. Future studies should explore the synergistic effects of ST1926 along with other therapeutic agents targeting these pathways to potentially identify enhanced therapeutic approaches.

## Data Availability

The mass spectrometry proteomics data have been deposited in the Data Dryad Repository and are available via the following link: http://datadryad.org/stash/share/a1bjlK_VnFGmTedzBQrmq0w415Hr70OTZLaWXv5TiiA. (accessed 25 January 2025).

## Supplementary Materials

The following supporting information can be downloaded at https://www.mdpi.com/article/10.3390/ijms26104651/s1.
