# Supplementary material for: Exploring the Anti-Leukemic Effect of the Synthetic Retinoid ST1926 on Malignant T Cells: A Comprehensive Proteomics Approach
Source: Int J Mol Sci. 2025 May 13;26(10):4651. doi: 10.3390/ijms26104651 (PMC12111145; doi:10.3390/ijms26104651)
Supplement: Supplementary file 1 [file ijms-26-04651-s001.zip › Supplementary Information_IJMS.pdf]

Supplementary Information

Exploring the Anti-leukemic Effect of the Synthetic Retinoid ST1926 on Malignant T cells: A Comprehensive Proteomics Approach

Mona Goli[1†], Vishal Sandilya[1†], Botheina Ghandour[2], Hiba El Hajj[3], Firas Kobeissy[2,4], Nadine Darwiche[2], Yehia Mechref[1]*

[1]Chemistry and Biochemistry Department, Texas Tech University, Lubbock, TX, USA
[2]Department of Biochemistry and Molecular Genetics, American University of Beirut, Beirut, Lebanon
[3]Department of Experimental Pathology, Immunology and Microbiology, American University of Beirut, Beirut, Lebanon
[4]Center for Neurotrauma, Multiomics & Biomarkers, Department of Neurobiology, Morehouse School of Medicine, Atlanta, Georgia, USA

***Correspondence:**

Yehia Mechref, Ph.D.

Texas Tech University, Department of Chemistry and Biochemistry
Box 41061, Lubbock, TX 79409-1061, USA
Telephone: +1 (806) 834-8246
E-Mail: Yehia.Mechref@ttu.edu

†Equal Contribution

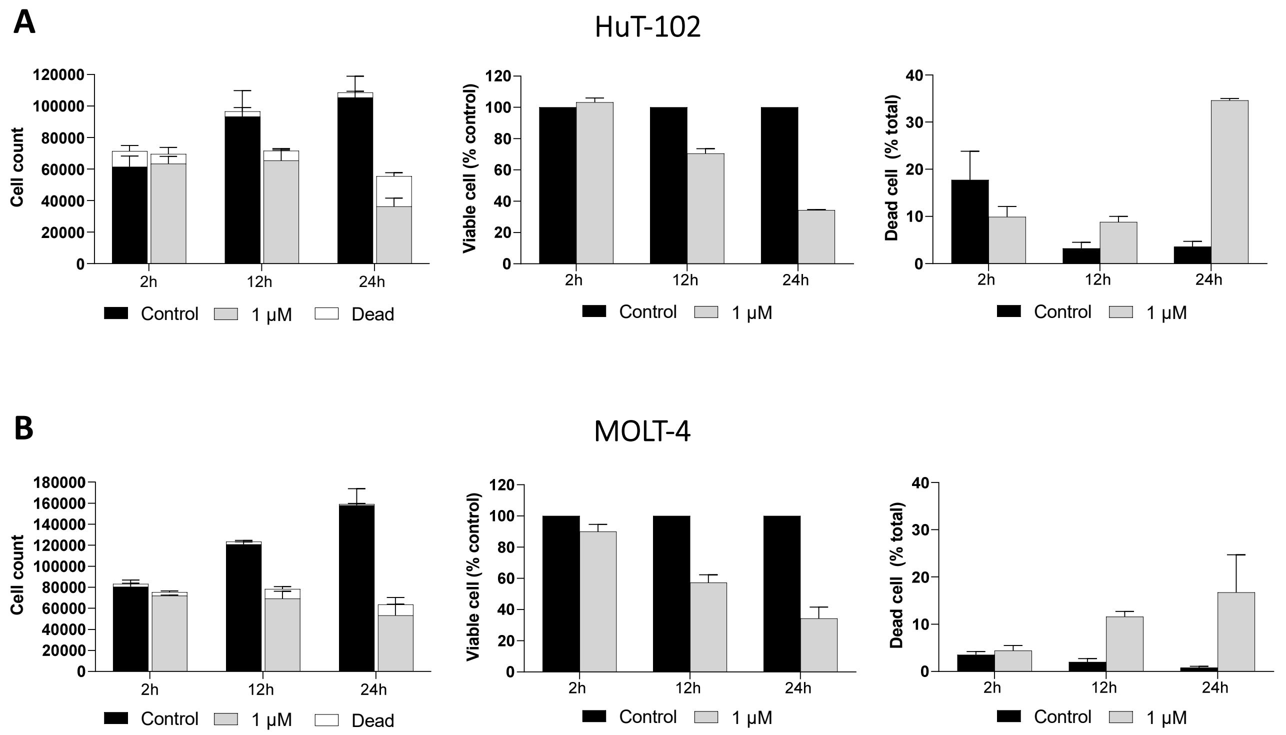

Figure S1. Testing the effect of ST1926 on HuT-102 and MOLT-4 cells growth, viability, and cell death. Cells were seeded in 24-well plates in triplicates and treated with 1 μM of ST1926 for 2, 12 and 24 hours. Trypan blue dye exclusion assay was performed to determine live/dead cells. A: HuT-102, B: MOLT-4. The bars represent the average cell count, cell viability (percent control), and cell death (percent total) of a two independent experiments with three wells per condition ± SD.

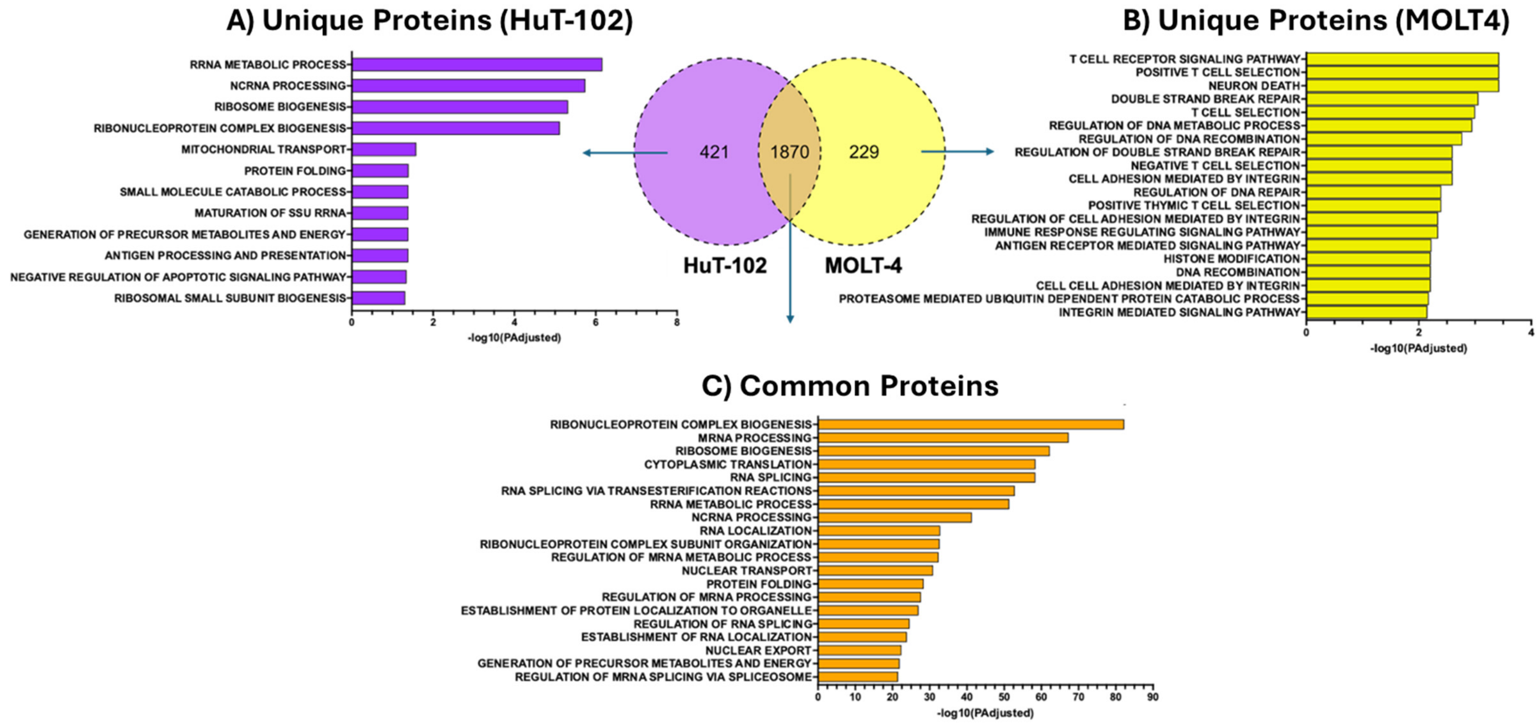

Figure S2. Comparison of total quantified proteins between Adult T-cell Leukemia/Lymphoma (HuT-102) and T-cell Acute Lymphoblastic Leukemia (MOLT-4) malignant T cells using Venn diagram. 1870 proteins were identified in common between the two cells. 421 unique proteins were identified in HuT-102, while 229 unique proteins were identified in MOLT-4. (A) The proteins unique to HuT-102 were predominantly involved in biological processes related to rRNA metabolic processing, ribosome biogenesis, and RNA processing pathways. (B) The unique proteins for MOLT-4 were heavily involved in T-cell-specific biological processes including T-cell receptor signaling and T-cell selection. (C) The biological processes for the 1870 common proteins between HuT-102 and MOLT-4

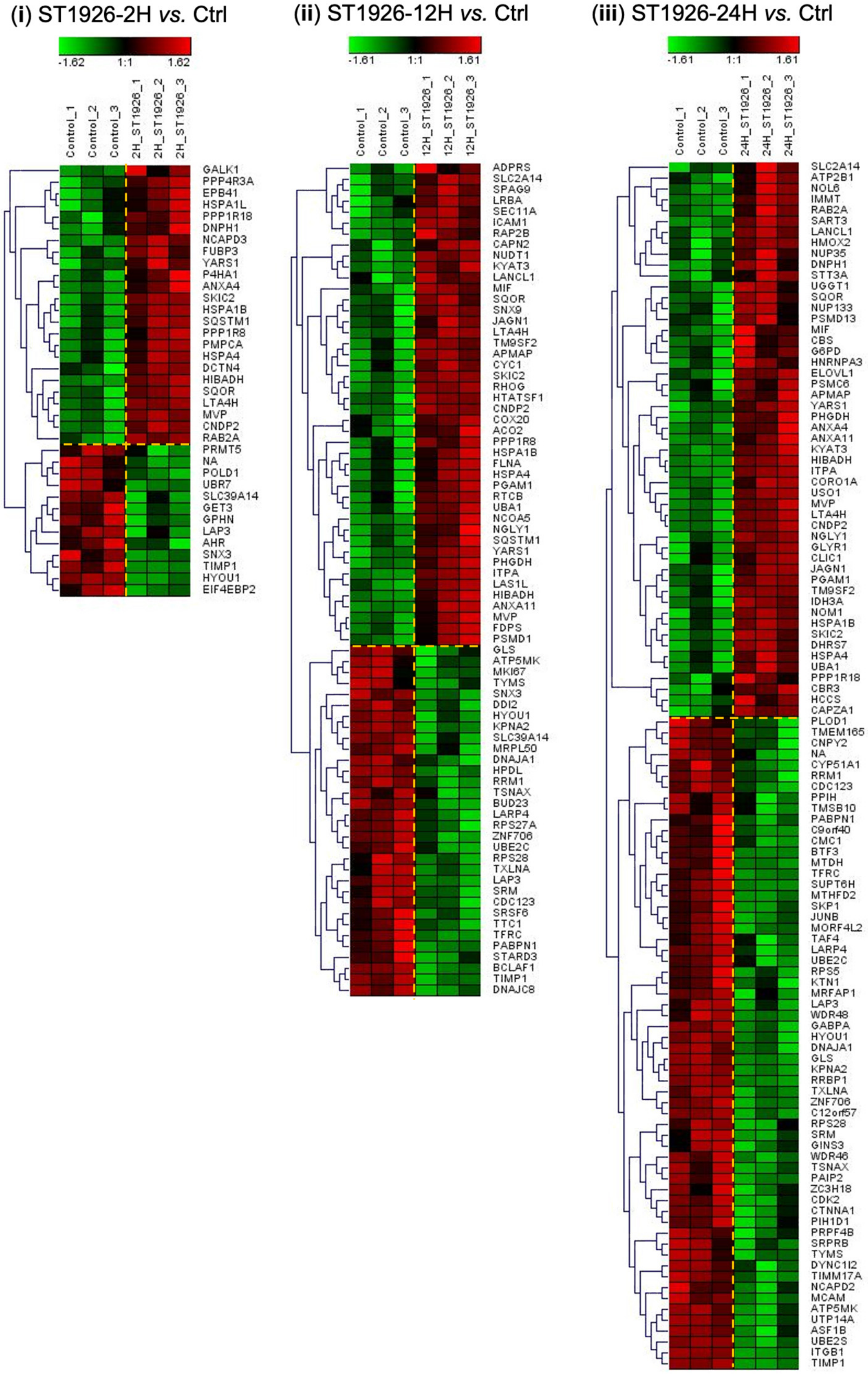

Figure S3. Hierarchical heatmap clustering visualizing the proteins with significant alterations in Adult T-cell Leukemia/Lymphoma cells (HuT-102) after treatment with ST1926 at (**i**) 2, (**ii**) 12, and (**iii**) 24 hours time points. Each row represents the abundance of a significant protein in two compared groups, while each column is the replicate of each group. The green color denotes a low relative abundance, while the red indicates a high relative abundance.

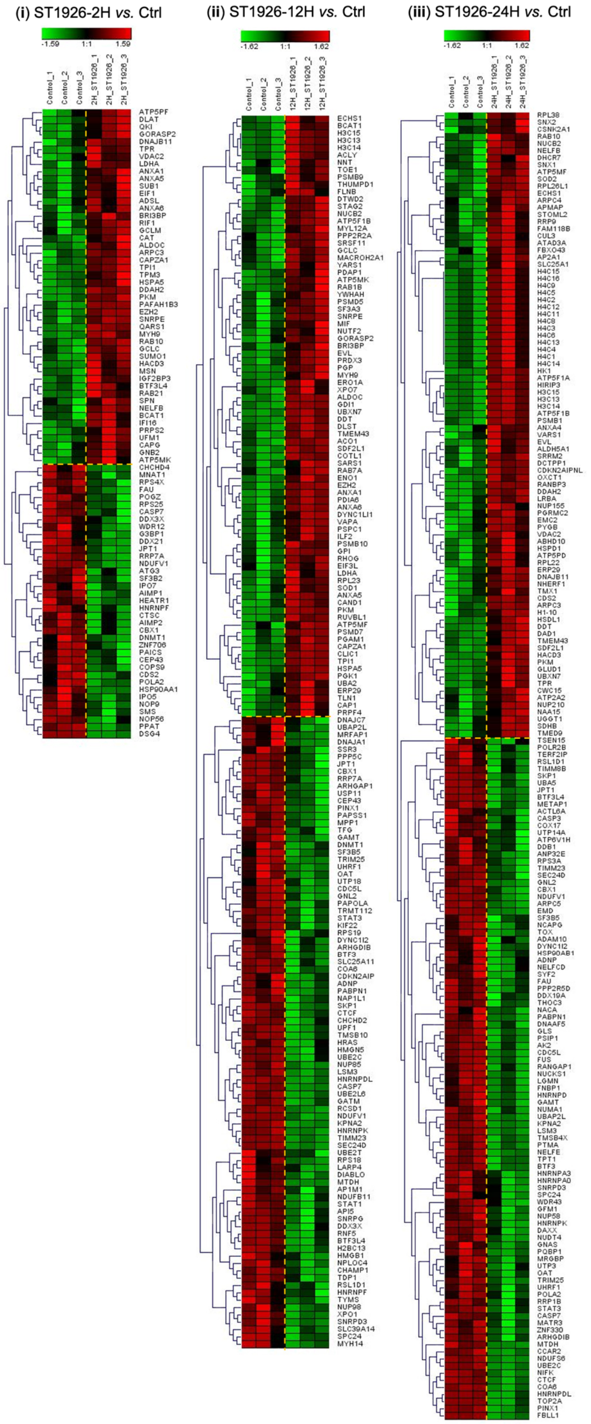

Figure S4. Hierarchical heatmap clustering visualizing the proteins with significant alterations in T-cell Acute Lymphoblastic Leukemia cells (MOLT-4) after treatment with ST1926 at (**i**) 2, (**ii**) 12, and (**iii**) 24 hours time points. Each row represents the abundance of a significant protein in two compared groups, while each column is the replicate of each group. The green color denotes a low relative abundance, while the red indicates a high relative abundance.

Goli *et al.*, Figure S5

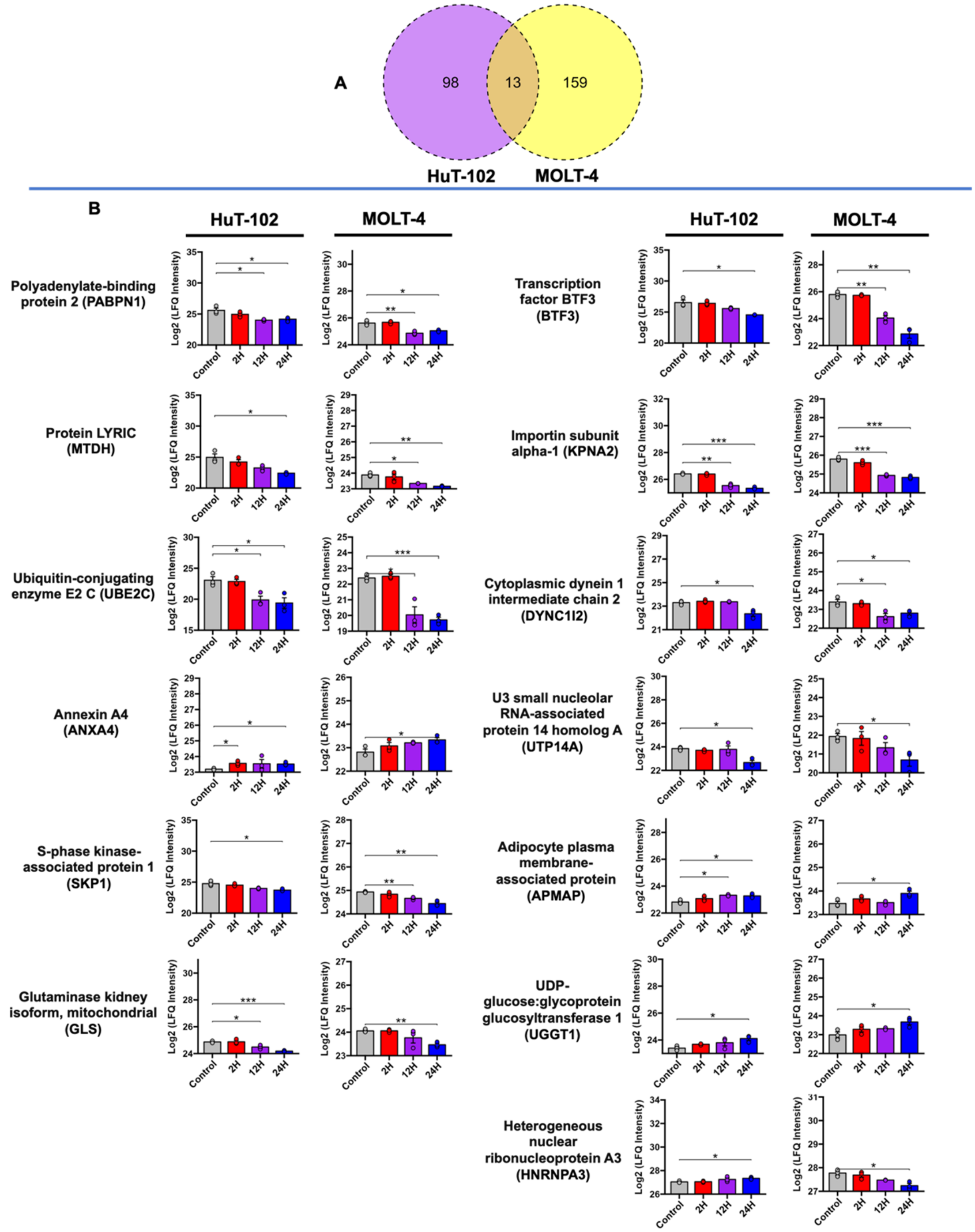

Figure S5. Comparative protein expression upon ST1926 treatment of Adult T-cell Leukemia/Lymphoma (HuT-102) and T-cell Acute Lymphoblastic Leukemia (MOLT-4) malignant T cells. (**A**) Venn diagram representing the unique and common differentially expressed proteins at 24 hours post-treatment with ST1926; (**B**) Dot plots demonstrating the expression of 13 common differentially expressed proteins among HuT-102 and MOLT-4malignant T cells at the indicated time points. *, *p*-value < 0.05, **, *p*-value < 0.01; ***, *p*-value < 0.001.

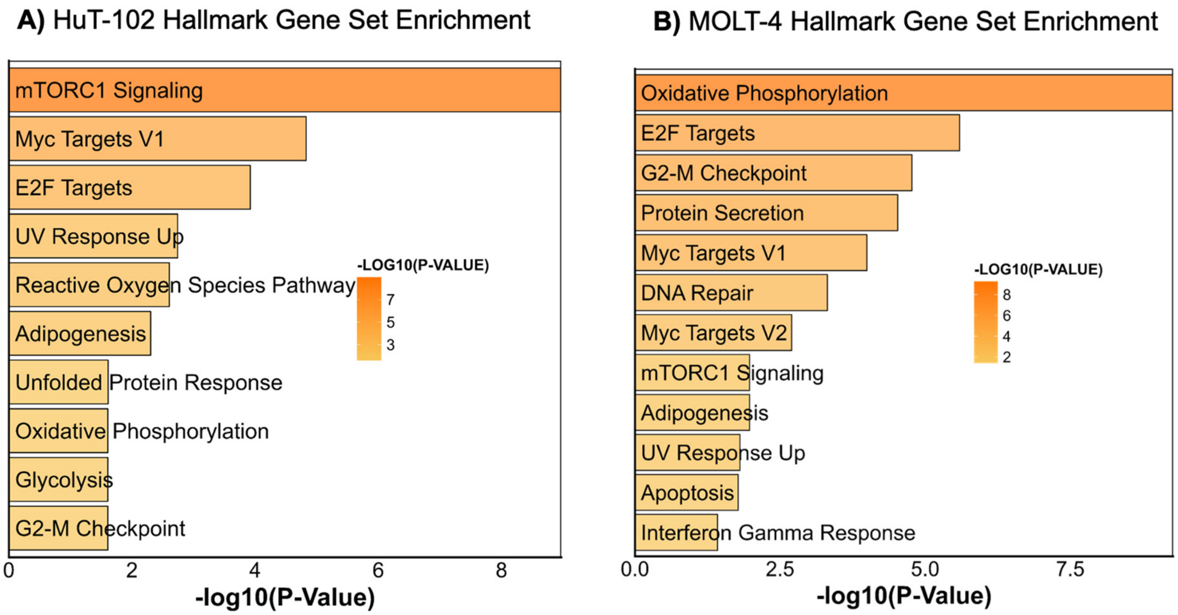

Figure S6. Hallmark gene set enrichment analysis of the significantly expressed proteins in Adult T-cell Leukemia/Lymphoma (HuT-102) (**A**) T-cell Acute Lymphoblastic Leukemia (MOLT-4) cells (**B**) negative malignant T cells after 24 hours of treatment with ST1926.

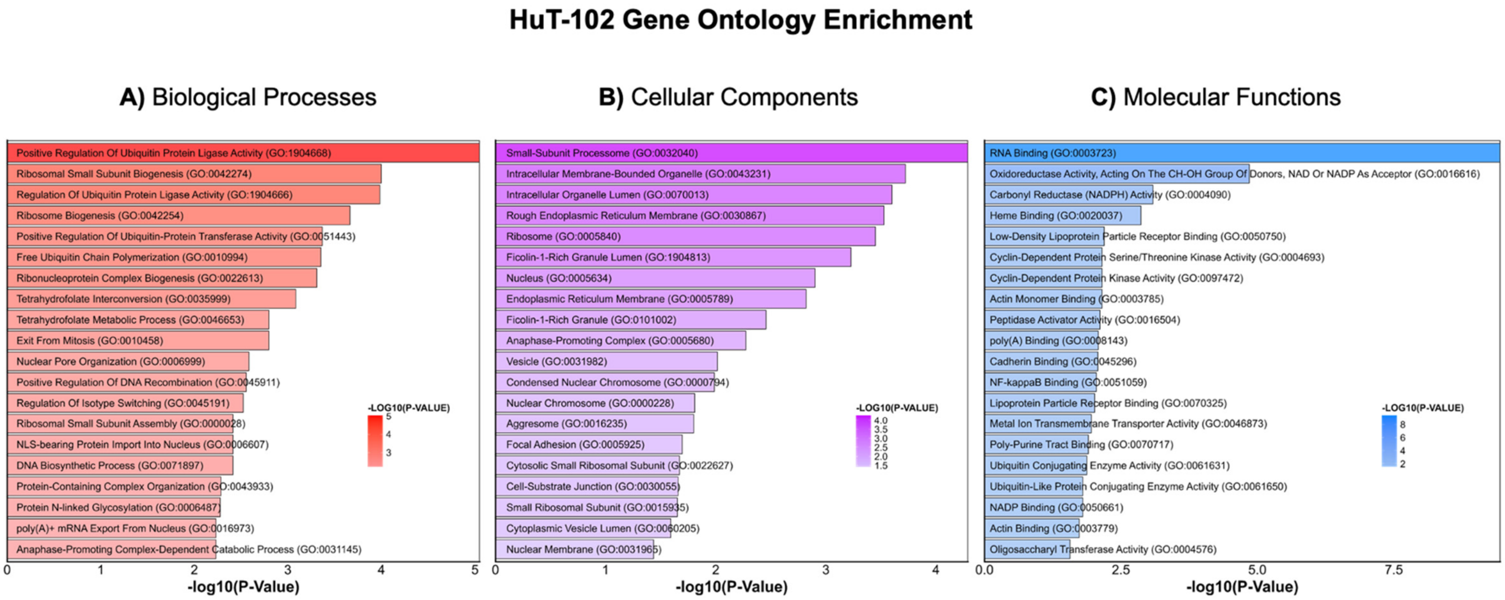

Figure S7. Gene Ontology Enrichment in Adult T-cell Leukemia/Lymphoma cells (HuT-102) at 24 hours treatment with ST1926.

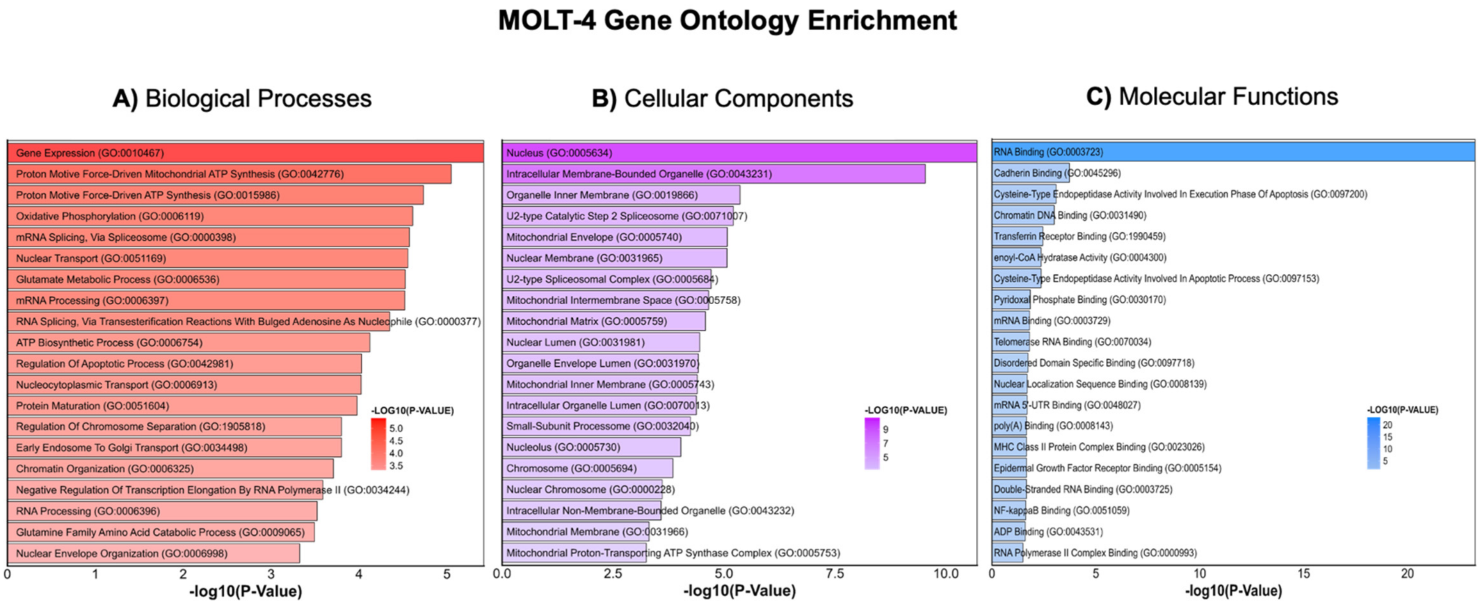

Figure S8. Gene Ontology Enrichment of T-cell Acute Lymphoblastic Leukemia cells (MOLT-4) at 24 hours treatment with ST1926.

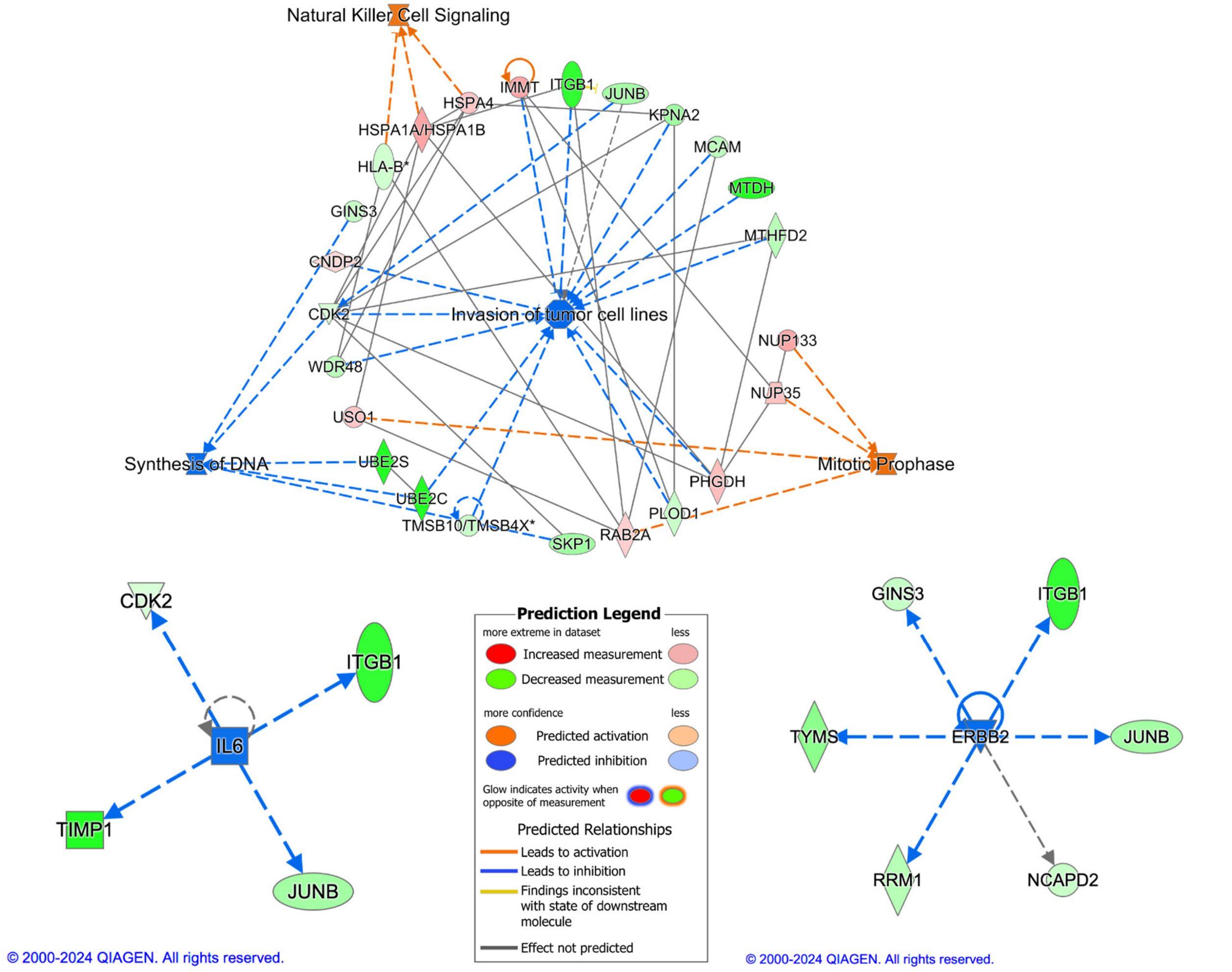

Figure S9. Ingenuity pathway analysis (IPA) for differentially expressed protein in Adult T-cell Leukemia/Lymphoma cells (HuT-102) after 24 hours of treatment with ST1926.

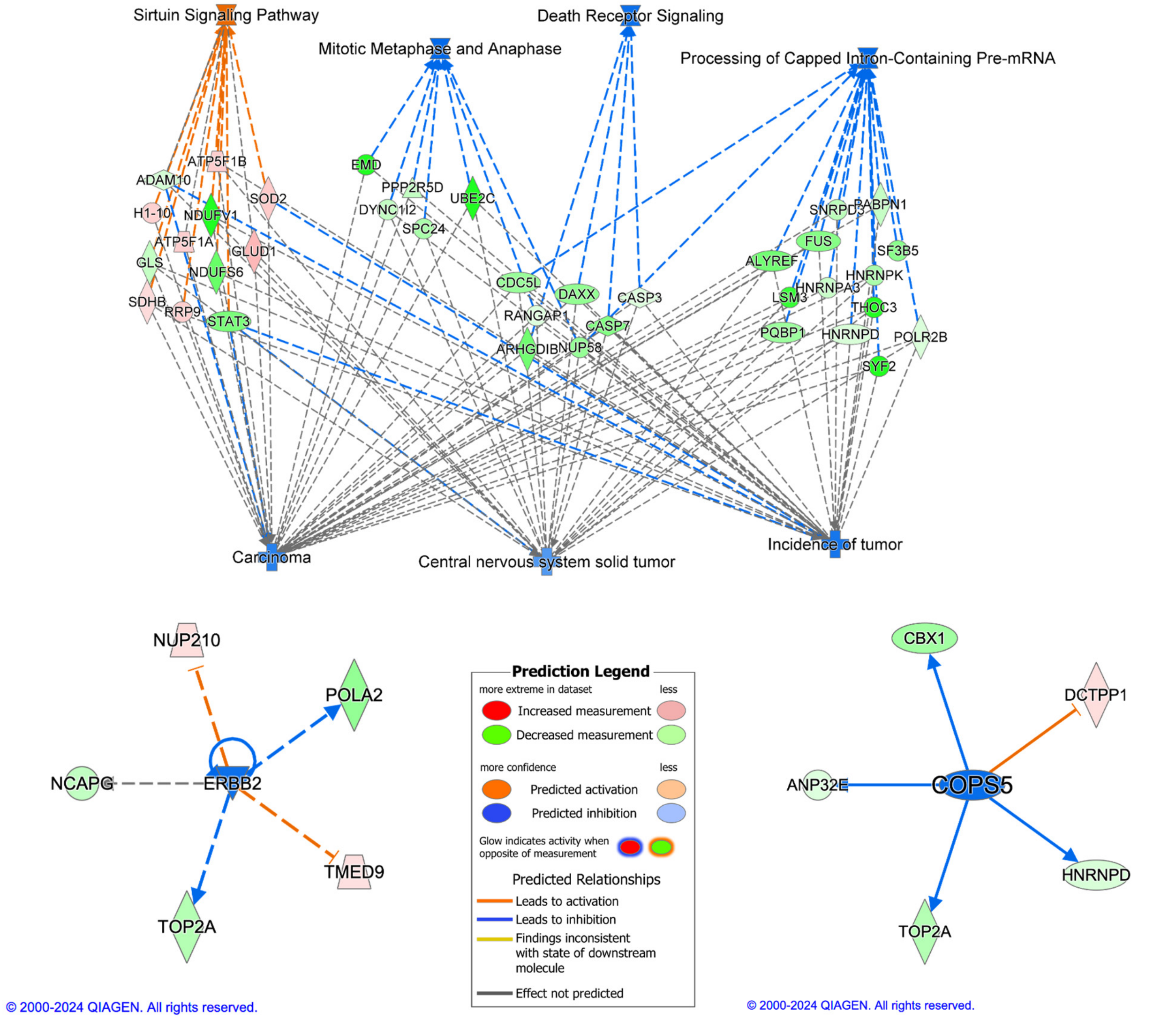

Figure S10. Ingenuity Pathway Analysis (IPA) for differentially expressed protein in T-cell Acute Lymphoblastic Leukemia cells (MOLT-4) after 24 hours of treatment with ST1926.
